# Calcitonin gene-related peptide promotes proliferation and inhibits apoptosis in endothelial progenitor cells via inhibiting MAPK signaling

**DOI:** 10.1186/s12953-018-0146-4

**Published:** 2018-11-14

**Authors:** Jianqun Wu, Song Liu, Zhao Wang, Shenghui Ma, Huan Meng, Jijie Hu

**Affiliations:** 1 Department of Spine Surgery, Huadu District People’s Hospital, Guangzhou, Guangzhou, 510800 Guangdong Province China; 2 Department of Orthopedics, The Third Affiliated Hospital of Guangzhou Medical University, Guangzhou City, 510150 Guangdong Province China; 3 Department of Orthopedics and Traumatology, Nanfang Hospital, Southern Medical University, Guangzhou City, 510515 Guangdong Province China

**Keywords:** Calcitonin gene-related peptide, Endothelial progenitor cells, Proliferation, Apoptosis, MAPK signalling pathway

## Abstract

**Background:**

Calcitonin gene-related peptide (CGRP) contributes to bone formation by stimulating bone marrow stromal cell (BMSC) proliferation and differentiation. However, the proliferative and apoptotic effects of CGRP on bone marrow-derived endothelial progenitor cells (EPCs) have not been investigated.

**Methods:**

We tested the effects of CGRP on EPC proliferation and apoptosis by Cell Counting Kit-8, flow cytometry, and studied the effects of CGRP on the expression of proliferation- and apoptosis-related markers in EPCs and the underlying mitogen-activated protein kinase (MAPK) signalling pathway by quantitative polymerase chain reaction and western blotting.

**Results:**

We detected EPC markers (CD34, CD133 and VEGFR-2) in 7-day cultures and found that CGRP (10^− 10^–10^− 12^ M) promoted the proliferation of cultured EPCs, with a peak increase of 30% at 10^− 10^ M CGRP. CGRP also upregulated the expression of proliferation-associated genes, including cyclin D1 and cyclin E, and increased the percentages of G2/M-phase and S-phase cells after incubation 72 h. CGRP inhibited serum deprivation (SD)-induced apoptosis in EPCs after 24 and 48 h and downregulated the expression of apoptosis-related genes, including caspase-3, caspase-8, caspase-9 and Bax. Phosphorylated (p-)ERK1/2, p-p38 and p-JNK protein levels in EPCs treated with CGRP were significantly lower than those in untreated EPCs. Pre-treatment with the calcitonin receptor-like receptor (CRLR) antagonist CGRP8–37 or a MAPK pathway inhibitor (PD98059, SB203580 or SP600125) completely or partially reversed the pro-proliferative and anti-apoptotic effects and the reduced p-ERK1/2, p-p38 and p-JNK expression induced by CGRP.

**Conclusion:**

Our results show that CGRP exerts pro-proliferative and anti-apoptotic effects on EPCs and may act by inhibiting MAPK pathways.

**Electronic supplementary material:**

The online version of this article (10.1186/s12953-018-0146-4) contains supplementary material, which is available to authorized users.

## Introduction

Studies in recent decades have revealed that angiogenesis has a close spatial and temporal association with bone development and remodelling [1, 2]. In 1997, endothelial progenitor cells (EPCs) were discovered and identified from peripheral blood, and many reports have confirmed that EPCs are incorporated into neovasculature and differentiate into mature endothelial cells [3–5]. Studies have reported more neovascularization and new bone formation due to EPC transplantation therapy in a segmental defect model compared with controls [6–9]. EPCs are mobilized, recruited and induced to proliferate by signals from bone regeneration sites, thus possibly contributing to neovascularization and new bone formation in fracture healing.

EPCs are being investigated for advanced bone metabolism therapies, and neuropeptides have an important role in osteogenic differentiation [10, 11]. Recent studies have shown that neuropeptide treatment increased the population size and the tube formation potential of endothelial cells and led to more effective assembly of endothelial cells into tubes [12]. In addition, neuropeptides such as calcitonin gene-related peptide (CGRP) have been shown to prevent circulating EPC senescence and to reverse Ang II-induced EPC senescence [13, 14]. CGRP is known to be a multifunctional neuropeptide that is primarily localized to the central and peripheral nervous systems, and it has been widely described to innervate and exert effects associated with the cardiovascular system [15]. However, there are numerous CGRP-immunoreactive nerve fibres in the periosteum, bone marrow, and epiphyseal trabecular bone [16, 17], and many reports suggest that CGRP has pivotal biological effects on the skeletal system in vivo [18, 19] and in vitro [20, 21]. Thus, two important and complex elements (EPCs and CGRP) are required for the progression of bone formation. Therefore, it is hypothesized that CGRP is a protective factor for EPCs to improve cell viability and number. Our study and previous studies indicated that CGRP receptor, a G-protein-coupled calcitonin receptor-like receptor (CRLR), is expressed in EPCs [22, 23]. More importantly, CGRP was confirmed to stimulate human endothelial cell proliferation in a concentration-dependent manner [22]. Zhou et al. showed an inverse correlation between the number of senescent EPCs and plasma CGRP levels and the CGRP mRNA expression in EPCs [14]. However, few studies have reported the process and molecular mechanisms by which CGRP affects EPC proliferation and apoptosis, which are indispensable for maintaining EPC numbers.

MAPKs (mitogen-activated protein kinases) are ancient and central signal transducers that include three subgroups: extracellular signal regulated kinase (ERK), c-Jun N-terminal kinase (JNK), and p38 signalling families [24]. MAPKs regulate a variety of cellular processes, including EPC apoptosis [25] and proliferation [26–28], and each MAPK family contributes distinctly to pro- and anti-apoptotic pathways in other cells [29, 30]. Activation of p38 MAPKs increases the number of circulating EPCs [25]; however, p38 activation but not ERK activation contributes to reducing the number of EPCs ex vivo [31]. EPC proliferation may be regulated by ERK phosphorylation, as evidenced by the significant inhibition of EPC proliferation induced by a selective ERK kinase inhibitor [27]. Therefore, we conducted an investigation to determine the effect of CGRP on EPCs and whether this effect is related to the MAPK signalling pathway.

## Materials and methods

### BM-derived EPC culture and characterization

Bone marrow-derived EPCs were prepared as previously described [32]. Briefly, bone marrow was isolated from the femurs of Sprague-Dawley rats (male, 80–100 g, Experimental Animal Center of Southern Medical University, Guangzhou, China). Mononuclear cells were isolated by density-gradient centrifugation with Ficoll (Hao Yang, TianJin, China) and cultured at a density of 1.0 × 10^5^ cells in fibronectin-coated 25-mm^2^ cell culture flasks (Corning, NY, USA) in a humidified incubator with 5% CO_2_ at 37 °C. Endothelial cell culture medium (EGM-2; Lonza) was supplemented with 100 U/mL penicillin, 100 μg/mL streptomycin, and 10% foetal bovine serum (FBS, Gibco, USA). The medium was changed in the first 24 h and then every 2–3 days. EPC purity was assessed by analysing the surface expression of CD34 (Novos, NBP1–44407), CD133 (AbNovo, PAB12663), and VEGFR2 (CST, 9698S) via flow cytometric assays and by immunofluorescence evaluation of double-positive DiI-Ac-LDL and FITC UEA-I staining after 7 days of culture, as these criteria have been classically used to characterize EPCs [6, 31, 32]. Third-passage cells were used for the following experiments. All rats received humane care in compliance with the National Institutes of Health Guide for the Care and Use of Laboratory Animals (NIH publication, revised 1996).

### Immunocytochemistry

To identify cultured cells as EPCs, adherent cells in 24-well plates were washed with PBS and supplemented with 200 μL DiI-ac-LDL (10 μg/mL) for 4 h at 37 °C in the dark. Cells were then fixed in precooled 4% paraformaldehyde for 10 min and stained with 200 μl of FITC-UEA-1 (10 μg/mL) for 1 h at 37 °C. After the liquid was discarded and cells were washed 3 times with PBS, the cells were visualized with a fluorescence microscope. To explore CRLR expression in the EPCs, the cells were fixed with 4% paraformaldehyde, incubated in anti-CRLR antibody (diluted 1:500) (Santa Cruz, sc-18,007) overnight at 4 °C, and then washed three times with PBS, followed by addition of Alexa Fluor 555-conjugated secondary antibodies at 37 °C for 1 h. Nuclear condensation and fragmentation was assessed using 4′-6′-diamidino-2-phenylindole (DAPI) staining by adding cells to 200 μl of DAPI solution (1 μg/mL) at room temperature for 10 min and analysing five random fields under a fluorescence microscope.

### Fluorescence-activated cell sorting

Cells were harvested at a density of 3 × 10^6^ cells/ml and incubated in primary antibody at 4 °C for 30 min. The following anti-rat primary antibodies were used for fluorescence cytometry: anti-CD34, anti-CD133, and anti-VEGFR-2, followed by incubation with Alexa Fluor 555-conjugated secondary antibodies at 4 °C for 30 min. Cells were resuspended with 400 μL of staining buffer, and the EPC surface molecules were analysed by flow cytometry (*FACSCanto*™ *II*, Becton-Dickinson, USA).

### Cell treatments

The experiments were divided into two parts. The first part was designed to develop an initial understanding of the effects of CGRP (alpha type, Abcam, ab47101) on EPC proliferation and apoptosis. An equal volume of PBS was added to the control group, and 10^− 8^ M, 10^− 10^ M, and 10^− 12^ M CGRP (final concentration) was added to the experimental groups [22, 23, 33]. The optimal concentration was obtained by different detection methods. The second part of the experiment was performed to examine the influence of CGRP receptors and the MAPK pathway in mediating the effects of CGRP on EPC proliferation and apoptosis. EPCs were preincubated with the CRLR antagonist CGRP8–37 (10^− 6^ M, Abcam, UK) and one of three MAPK pathway inhibitors, PD98059 (EPK), SB203580 (p38), or SP600125 (JNK) (10^− 6^ M, BD Biosciences, USA), for 30 min. Then, the peak effective dose of CGRP was added.

### EPC viability assay

The viability of EPCs was assessed using a Cell Counting Kit-8 (CCK-8, Dojindo, Japan) assay. Briefly, EPCs (5 × 10^3^ cells/well) were seeded in a 96-well plate (*n* = 6, in triplicate) with 100 μl of high-glucose DMEM in the presence (for proliferation) or absence (fetal serum deprivation [SD]-induced apoptosis) of 10% FBS. The culture medium containing the appropriate stimulus was changed daily. After 12, 24 and 48 h (for apoptosis assays) or 1, 3, 5, and 7 days (for proliferation assays), CCK-8 reagent (10 μL/100 μL) was added to the EPC cultures, and the cultures were incubated for an additional 2 h at 37 °C in 5% CO_2_. The reaction was stopped, and the optical density was determined at 450 nm using a SpectraMax_M5 plate reader (Molecular Devices, USA).

### Cell cycle analysis

Cell cycle assays were performed with a Cell Cycle Detection Kit (KeyGEN Biotech, Nanjing, China) using flow cytometry. Briefly, EPCs in each group at each time point were harvested and fixed in 70% precooled ethanol overnight at 4 °C. Then, 100 μl of RNase A and 500 μl of propidium iodide (PI) were added at 37 °C for 30 min. DNA content was measured by flow cytometry .

### Apoptosis analysis

Apoptosis was determined using an Annexin V-FITC Apoptosis Detection Kit (KeyGEN Biotech, Nanjing, China). Cells were trypsinized (Ca^2+^ free), washed three times with PBS, and resuspended in binding buffer. They were then incubated with 5 μl of annexin V-FITC and 5 μl of PI solution in the dark for 10 min and examined by flow cytometry (*FACSCanto*™ *II*, Becton-Dickinson, USA).

### Western blotting

Cells were cultured as described above. EPCs (1 × 10^6^ cells) were then harvested and lysed in RIPA buffer, followed by high-speed centrifugation for 5 min at 4 °C*.* Proteins (20–30 μg) were separated by sodium dodecyl sulphate in 10% polyacrylamide gels and transferred onto polyvinylidene difluoride membranes. The membranes were incubated with primary antibodies against p-ERK1/2, p-p38, p-JNK (Additional files 1 and 2) (1:250 dilution; Cell Signaling Technology; 1213/10, 1202/1, and 1496/10, respectively), cleaved caspase-3 (1:500 dilution; Abcam, ab49853), and β-catenin (1:250 dilution; Santa Cruz). Densitometric analysis was performed using ImageJ, and protein expression was normalized to that of β-actin and the control group.

### Quantitative real-time PCR

Total RNA from EPCs cultured in 25-mm^2^ cell culture flasks was extracted with TRIzol reagent (Invitrogen) according to the manufacturer’s instructions. The purity and concentration were determined spectrophotometrically. Real-time PCR reactions were performed using SYBR Green assays (Applied Biosystems, USA). Thermal cycling and fluorescence detection were performed with a StepOnePlus Real-Time PCR System (Applied Biosystems, USA). Primer sequences for cyclin D1, cyclin E, caspase-3, caspase-8, caspase-9, Bcl2, and Bax were used for PCR amplification (Table 1), and expression levels were compared with that of β-actin using the ΔCt method. All primers for qRT-PCR were designed using Primer Express software (ABI).

**Table 1:** Primers for qPCR

Gene	Sequence	Length (bp)	Accession number
cyclin D1	TGAAGTTCATTTCCAACCCA	150 bp	NM_171992.4
	AGTCCGGGTCACACTTG		
cyclin E	GACACAGCTTCGGGTCTG	137 bp	NM_001100821.1
	TTTGCCTTCCTTTTTCTGGA		
Caspase 3	CTGGACTGCGGTATTGAGAC	104 bp	XM_006253130.2
	CATGGGATCTGTTTCTTTGC		
Bcl-2	TGTGGCCTTCTTTGAGTTCG	149 bp	NM_016993.1
	CATCCCAGCCTCCGTTATCC		
Bax	TGCTACAGGGTTTCATCCAGG	114 bp	NM_017059.2
	TGAGACACTCGCTCAGCTTCTTG		
caspase 8	CCTATGCCACCTAGTGATTA	94 bp	NM_022277.1
	TATAAAACACCGGAGGTCAG		
caspase 9	CTGCGTCTCATCAAAGTTTC	90 bp	NM_031632.1
	GACAGTCCTTTTGCCTAAGA		

β-actin was used as the internal control for normalizing the gene expression results, and all results were confirmed by repeating the experiments 3 times.

### Statistical analysis

The results are expressed as the mean ± SEM. After testing for normality and equal variance, differences between two groups were analysed using Student’s t-test. Groups were evaluated by one-way ANOVA followed by the Bonferroni test. A *P* value of < 0.05 was considered statistically significant.

## Results

### Characterization of rat BM-derived EPCs

After 7 days of culture, colonies that originated from adherent cells emerged with a cobblestone appearance under an inverted microscope. A combination of stem cell and endothelial cell markers is commonly used for identifying EPCs via fluorescence cytometry analyses. The analysis revealed that EPCs expressed not only the haematopoietic stem cell marker CD34 but also endothelial cell antigens such as CD133 and VEGFR. The cells were positive for ac-LDL uptake and UEA-1 staining, which were identified by immunocytochemistry. After replating, the third-passage cells appeared spindle-shaped and formed a monolayer with a homogenous appearance. Immunocytochemical staining demonstrated the presence of CRLR in the EPCs (identification shown in another article) Additional file 3.

### Effect of CGRP on EPC proliferation

**Fig. 1:**
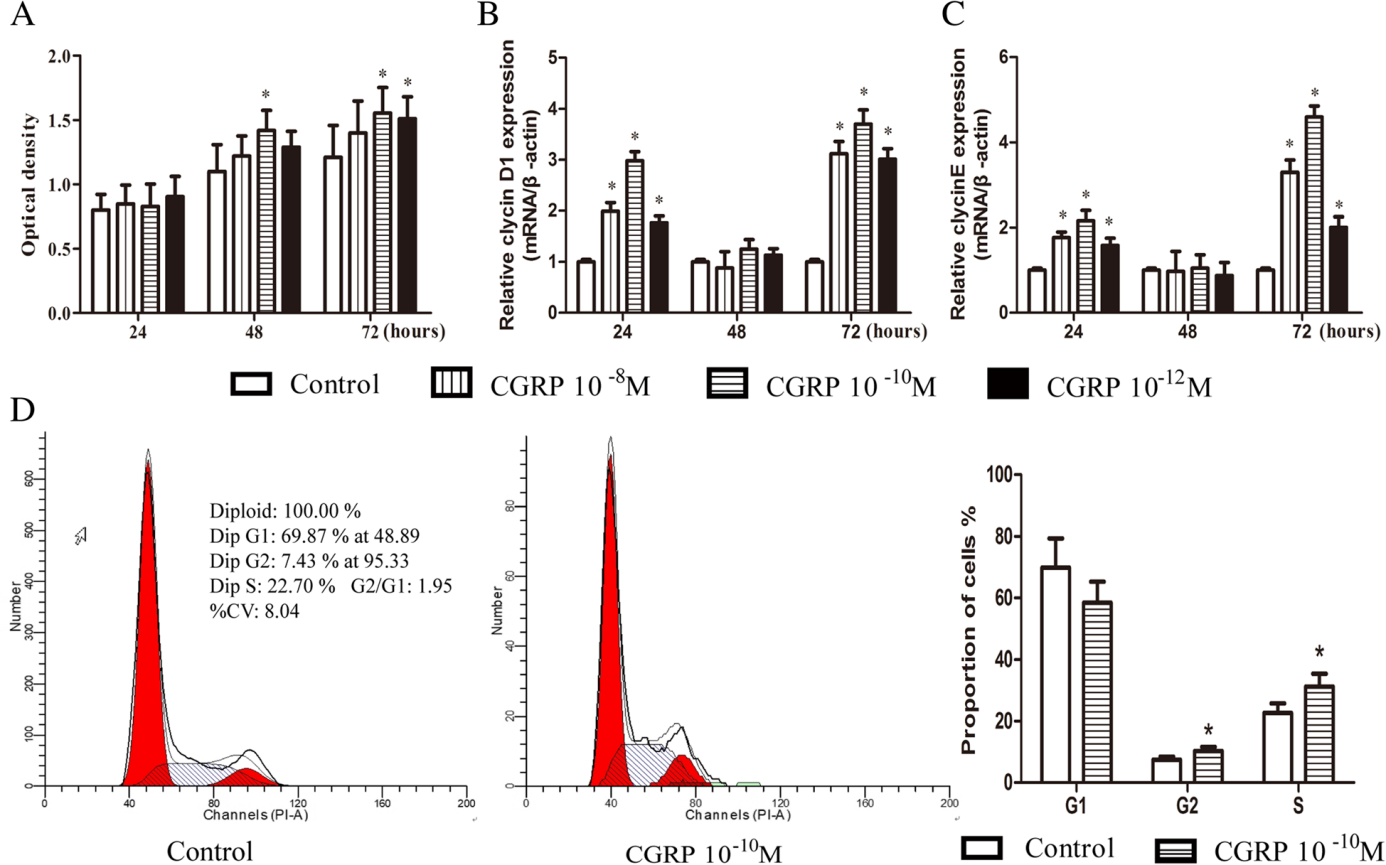
CGRP stimulated the proliferation of EPCs in dose- and time-dependent manners. After 24 h, 48 h, and 72 h of incubation with or without a series of concentrations CGRP (10^− 8^–10^− 12^ M) in high-glucose DMEM with 10% FBS, cell viability was assessed by the optical density via CCK-8 staining (**a**). Q-PCR was used to detect two proliferation-related genes, cyclin D1 (**b**) and cyclin E (**c**). Flow cytometry analysis of the cell cycle after 72 h of incubation (**d**). Data represent the means ± SEM of three experiments. * *p* < 0.05 compared with control

EPC viability was estimated in vitro using a CCK-8 assay. A dose-dependent increase in the mean optical density (*n* = 6) was observed from 24 to 72 h after stimulation with CGRP (Fig. 1a). At a CGRP concentration of 10^− 10^ M, the optical density was increased by 30 and 28% at 48 and 72 h, respectively, and no significant differences were identified among the four groups at 24 h (Fig. 1a). To further examine CGRP-induced EPC proliferation, two proliferation-related genes, cyclin D1 and cyclin E, were evaluated by q-PCR. CGRP (10^− 8^–10^− 12^ M) significantly increased cyclin D1 and cyclin E gene expression in a dose- and time-dependent manner, and the concentration of 10^− 10^ M appeared to be the most efficient (Fig. 1b, c). CGRP treatment at 10^− 8^, 10^− 10^ and 10^− 12^ M significantly increased cyclin D1 expression by 1.99-, 2.98- and 1.77-fold, respectively, at 24 h and by 3.12-, 3.70-, and 3.00-fold, respectively, at 72 h, but cyclin D expression was not altered at 48 h (Fig. 1b). Cyclin E gene expression levels were 1.77-, 2.16-, and 1.50-fold higher than those of the untreated controls at 24 h and 3.30-, 4.60-, and 2.00-fold higher than those of the untreated controls at 72 h with CGRP treatment at 10^− 8^, 10^− 10^ and 10^− 12^ M, respectively. However, there was no difference in cyclin E gene expression at 48 h (Fig. 1c).

To determine the mechanism by which CGRP promoted cell proliferation, we analysed cell cycle distribution using flow cytometry. Significantly increased percentages of G2/M-phase cells and S-phase cells were observed in the 10^− 10^ M CGRP-treated group after 72 h of incubation, and a decrease in the G1 phase fraction was observed, although the decrease was not significant (Fig. 1d). These results indicated that CGRP accelerated cell cycle progression from G1 phase to S phase and promoted EPC proliferation.

### Effect of CGRP on EPC apoptosis

**Fig. 2:**
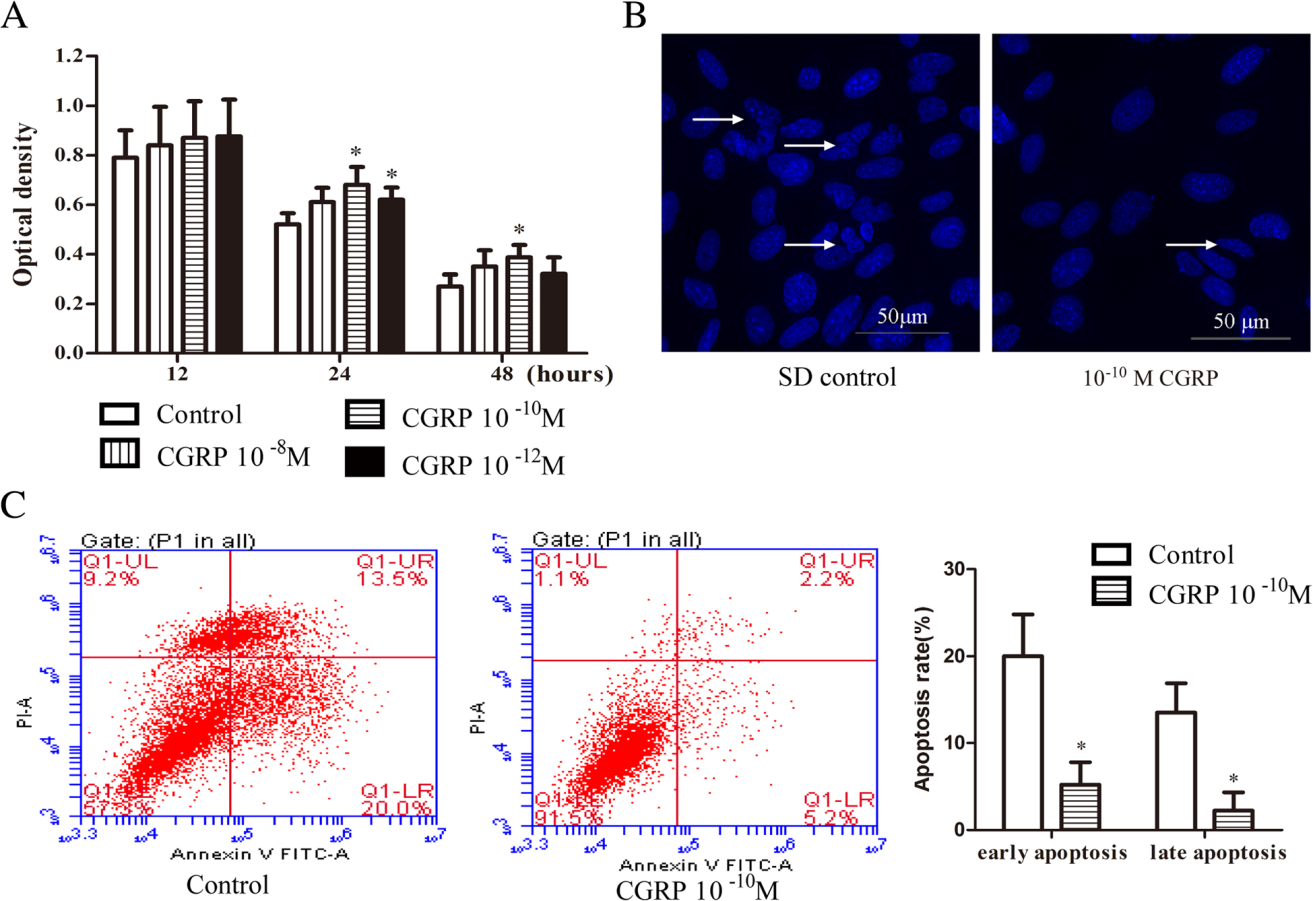
CGRP inhibited EPC apoptosis. After 12 h, 24 h, and 48 h of incubation with or without CGRP (10^− 8^–10^− 12^ M) in high-glucose DMEM during SD-induced apoptosis, cell viability was assessed by the optical density and by CCK-8 staining (**a**). The protective effects of 10^− 10^ M CGRP on the morphology of cell nuclei were evaluated by DAPI staining; the arrow indicates a cell nucleus that has undergone condensation and fragmentation (**b**). Cell apoptosis of control EPCs and EPCs stimulated with 10^− 10^ M CGRP after 48 h was analysed by flow cytometry analysis (**c**). Data represent the means ± SEM of three experiments. * *p* < 0.05 compared with control

To further evaluate the role of CGRP in EPCs, we cultured cells in the presence of CGRP (10^− 8^–10^− 12^ M) or an equal volume of PBS as a control during SD-induced spontaneous apoptosis for 12, 24, and 48 h. While treatment with CGRP significantly increased cell viability (Fig. 2a; 67 and 62% in 10^− 10^ and 10^− 12^ M CGRP-treated cells, respectively, vs. 52% in untreated cells at 24 h; 37% in 10^− 10^ M CGRP-treated cells vs 27% in untreated cells at 48 h), the CGRP protective effect was not observed at the 12-h time point. FBS-free medium induced apoptosis within 48 h; DAPI staining showed that many nuclei of the control cells appeared condensed and fragmented (Fig. 2b), but this morphology was reduced in the 10^− 10^ M CGRP-treated cells. Flow cytometry analysis showed that the early and late apoptosis rates of cells in the 10^− 10^ M CGRP-treated group were markedly decreased after 48 h of SD compared with the apoptosis rates of control cells (Fig. 2c). Collectively, these results suggested that CGRP suppressed SD-induced apoptosis in vitro.

**Fig. 3:**
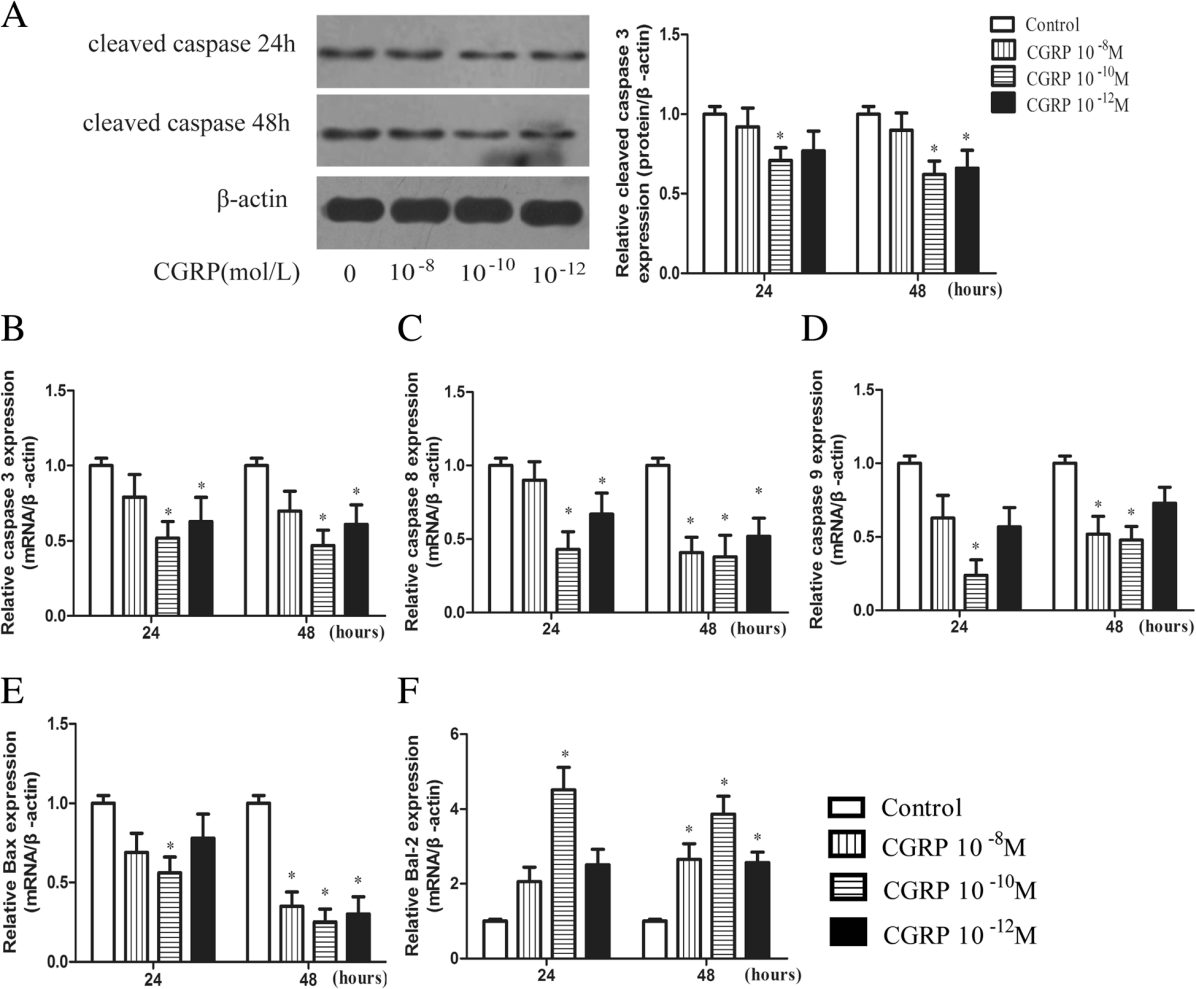
CGRP reduced the levels of apoptotic molecules in EPCs. Cells were serum starved for 24 h or 48 h with or without CGRP (10^− 8^–10^− 12^ M), and the expression level of caspase-3 protein was determined with Western blot (**a**). The transcriptional levels of apoptotic-related genes, including caspase-3, caspase-8, caspase-9, Bax and Bcl-2, were analysed by q-PCR (**b**-**f**). Data represent the means ± SEM of three experiments. * *p* < 0.05 compared with control

After cells were serum starved in the presence of CGRP (10^− 8^–10^− 12^ M) or an equal volume of PBS for 24 or 48 h, caspase-3 protein levels were quantitatively analysed by western blot, and apoptosis-related genes, including caspase-3, caspase-8, caspase-9, Bax and Bcl-2, were analysed by q-PCR. As shown in Fig. 3a, 10^− 10^ M CGRP treatment for 24 and 48 h significantly decreased caspase-3 protein levels compared with the control treatment; the relative caspase-3 protein expression level in the 10^− 10^ M CGRP-treated group was 0.71 ± 0.08 and 0.62 ± 0.09 after 24 and 48 h, respectively. The relative caspase-3 mRNA expression level in the 10^− 10^ M CGRP-treated group was 0.52 ± 0.11 and 0.47 ± 0.10 after 24 and 48 h, respectively, which was significantly different from that observed in the control group (Fig. 3b). Significant reductions in active caspase-3 protein and mRNA levels were also observed in EPCs after 48 h of 10^− 12^ M CGRP treatment (Fig. 3a, b). The mRNA expression of the apoptotic molecules caspase-8 and caspase-9 was significantly reduced by 10^− 10^ M CGRP after serum starvation for 24 and 48 h (Fig. 3c, d). Significant differences in caspase-8 mRNA expression were also observed for 10^− 12^ M CGRP at the 24 h and 48 h time points (Fig. 3c). Decreased Bax mRNA expression and increased Bcl-2 mRNA expression were clearly observed in the 10^− 10^ M CGRP-treated group at 24 h and in the three concentration groups at 48 h, indicating a higher Bcl-2/Bax ratio than in the control group (Fig. 3e, f). Taken together, these results indicate that CGRP downregulated apoptosis-related molecules, such as caspase-3, caspase-8, caspase-9 and Bax, and upregulated the anti-apoptotic molecule Bcl-2 in a dose-dependent manner after 24 h and 48 h of serum starvation.

### CRLR antagonist or MAPK pathway inhibitor treatment reduces the pro-proliferative and anti-apoptotic effects of CGRP on EPCs

**Fig. 4:**
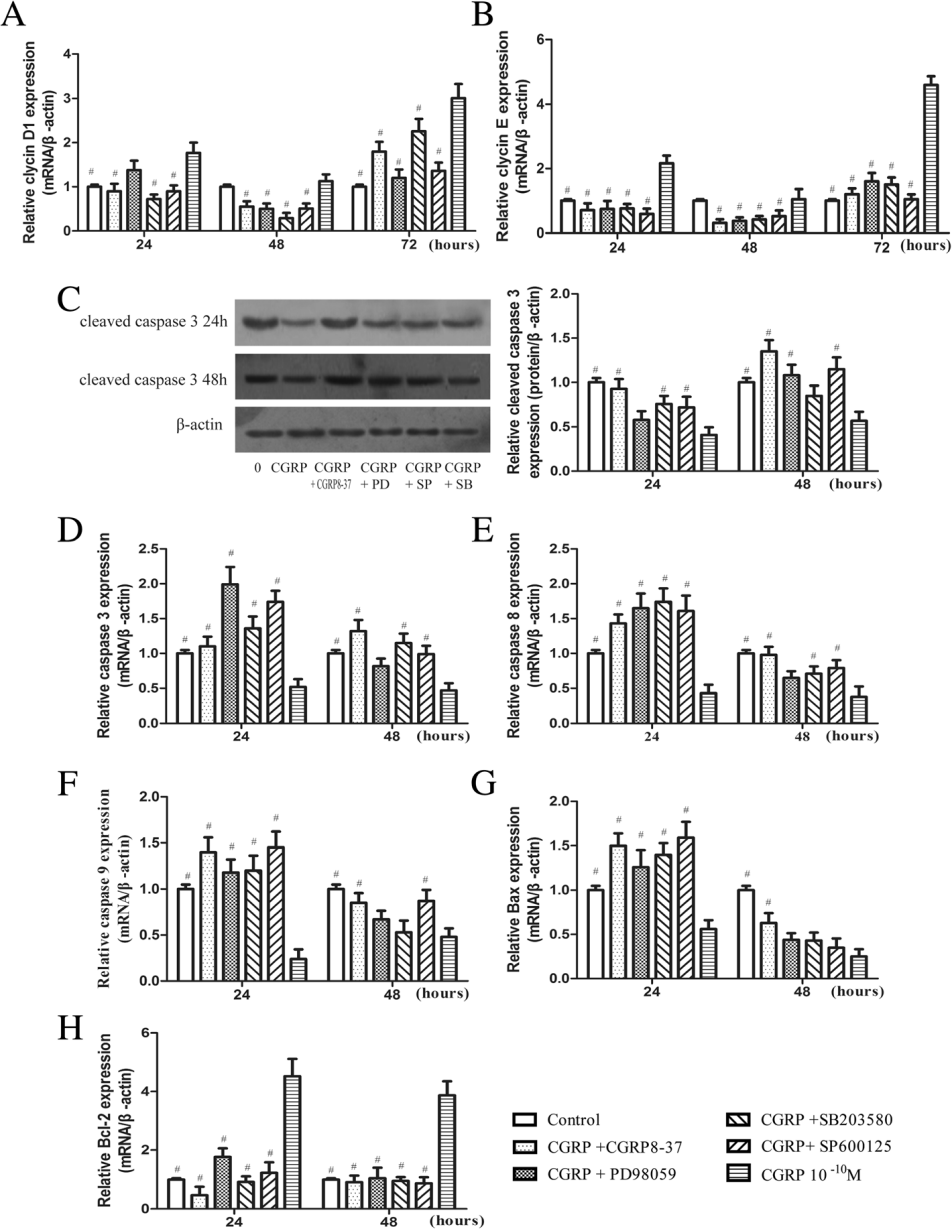
Effects of CGRP8–37, PD98059, SB203580, and SP600125 on EPC proliferation and apoptosis. Quantitative analysis of the proliferative target genes cyclin D1 and cyclin E (**a**, **b**) or apoptotic target genes caspase-3, caspase-8, caspase-9, Bax and Bcl-2 (**d**-**h**). The protein levels of cleaved caspase-3 were evaluated by Western blot (**c**). ^#^ *p* < 0.05 compared with the CGRP-only group

To determine whether CRLR or MAPK signalling plays a role in the promotion of EPC viability, we pretreated EPCs for 30 min with the CRLR antagonist CGRP8–37 (10^− 6^ M) or one of three MAPK pathway inhibitors, PD98059, SB203580, or SP600125 (10^− 6^ M), and then added 10^− 10^ M CGRP (at the peak effective dose, as described above). As shown in Fig. 4, the majority of the experimental groups significantly differed from the control groups. We found higher cyclin D1 and cyclin E mRNA expression levels in the 10^− 10^ M CGRP-treated cells than in the control cells (Fig. 4a, b), whereas CGRP8–37 and the MAPK pathway inhibitors prevented upregulation of these mRNAs (Fig. 4a, b). The results revealed that 10^− 10^ M CGRP suppressed caspase-3 protein and gene expression; however, the gene and protein expression of caspase-3 were no longer downregulated when cells were pretreated with the inhibitors (Fig. 4c, d) CGRP8–37 (0.93 ± 0.11 and 1.35 ± 0.13 relative protein levels; 1.10 ± 0.14 and 1.32 ± 0.16 relative mRNA levels after 24 and 48 h, respectively), PD98059 (0.58 ± 0.10 and 1.08 ± 0.12 relative protein levels; 2.00 ± 0.25 and 0.82 ± 0.11 relative mRNA levels after 24 and 48 h, respectively), SB203580 (0.76 ± 0.09 and 0.85 ± 0.11 relative protein levels; 1.36 ± 0.17 and 1.15 ± 0.13 relative mRNA levels after 24 and 48 h, respectively), SP600125 (0.72 ± 0.12 and 1.15 ± 0.13 relative protein levels; 1.74 ± 0.16 and 0.99 ± 0.12 relative mRNA levels after 24 and 48 h, respectively). Similar results were observed for caspase-8, caspase-9 and Bax mRNA (Fig. 4e, f, g). Bcl-2 is an anti-apoptotic molecule, and CGRP8–37 and MAPK pathway inhibitors blocked the Bcl-2 mRNA upregulation induced by CGRP (Fig. 4h). Thus, CRLR or MAPK signalling inhibitors may reverse CGRP-mediated EPC protection in vitro by regulating the proliferation target genes cyclin D1 and cyclin E and the apoptosis target genes caspase-3, caspase-8, caspase-9, Bax and Bcl-2.

### CGRP decreases MAPK pathway protein phosphorylation during EPC proliferation

**Fig. 5:**
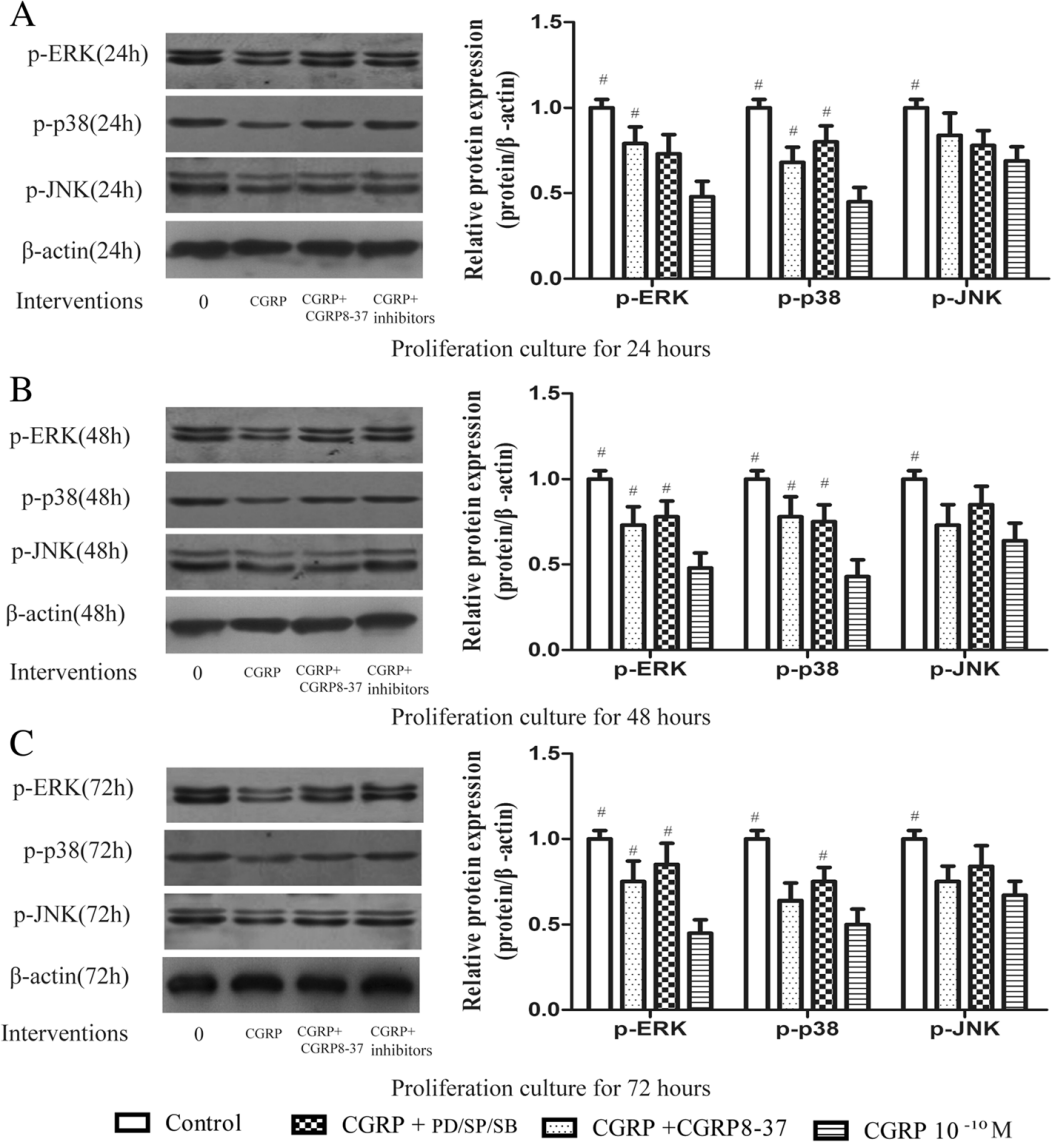
ERK1/2, p38, and JNK phosphorylation during EPC proliferation as assessed by Western blot analysis. Cells were incubated in complete DMEM with CGRP or CGRP combined with CGRP8–37, PD98059, SB203580, or SP600125 and incubated for 24 h, 48 h, or 72 h. ^#^ *p* < 0.05 compared with the CGRP-only group

We then used western blot analysis to examine whether CGRP alters MAPK pathway protein phosphorylation during EPC proliferation. The results showed that compared with untreated cells, 10^− 10^ M CGRP-treated cells showed remarkably reduced ERK1/2, p38, and JNK phosphorylation at 24, 48, and 72 h (Fig. 5a-c). Moreover, preincubation with CGRP8–37, PD98059, SB203580, or SP600125 reversed the CGRP-induced downregulation of the three phosphorylated proteins (p-ERK1/2, p-p38, and p-JNK), although statistical significance was observed in only a portion of the results; some of the results showed slightly higher phosphorylated protein levels in the inhibitor-treated groups than in the group treated with CGRP alone (Fig. 5a-c). In contrast to CGRP-treated cells, CGRP8–37-pretreated cells showed upregulated expression of p-ERK1/2, p-p38, and p-JNK (by 1.65-, 1.51- and 1.22-fold, respectively, at 24 h; 1.52-, 1.81- and 1.14-fold, respectively, at 48 h; and 1.67-, 1.28- and 1.20-fold, respectively, at 72 h). p-ERK1/2 expression was increased by 1.52-, 1.63- and 1.89-fold at 24, 48, and 72 h, respectively, in the PD98059-pretreated group compared with the CGRP-treated group. p-p38 expression was increased by 1.78-, 1.74- and 1.50-fold at 24, 48, and 72 h, respectively, in the SB203580-pretreated group compared with the CGRP-treated group. p-JNK expression was increased by 1.13-, 1.33- and 1.25-fold at 24, 48, and 72, respectively, in the SP60012-pretreated group compared with the CGRP-treated group.

### CGRP decreased MAPK pathway protein phosphorylation during SD-induced EPC apoptosis

**Fig. 6:**
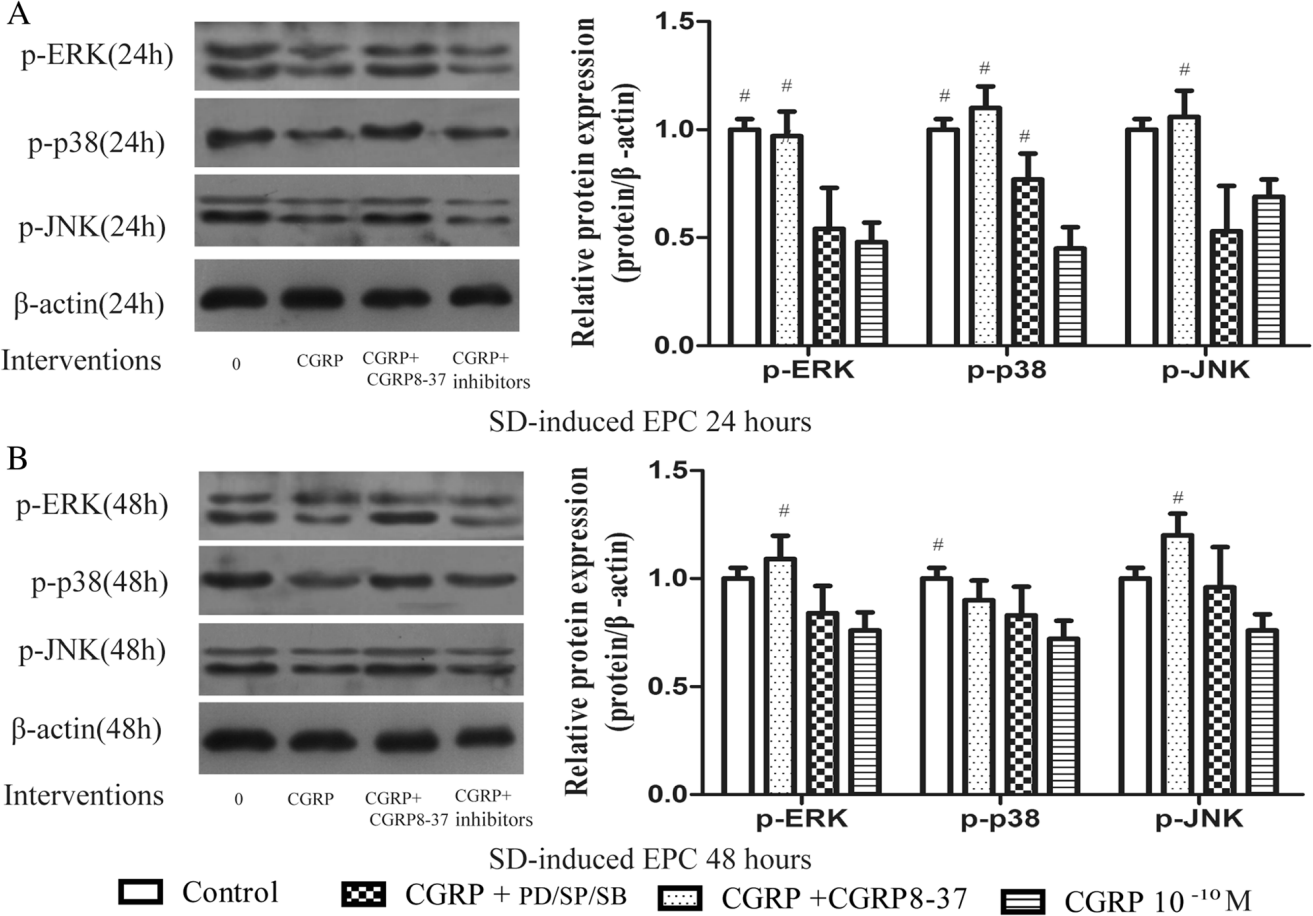
p-ERK1/2, p-p38, and p-JNK protein levels during EPC apoptosis as assessed by Western blot analysis. Cells were serum starved for 24 h or 48 h with CGRP or CGRP combined with CGRP8–37, PD98059, SB203580, or SP600125. ^#^ *p* < 0.05 compared with the CGRP-only group

When the same experimental groups were used to investigate SD-induced EPC apoptosis, similar findings were observed. CGRP inhibited p-ERK1/2, p-p38, and p-JNK protein levels according to western blot analysis, and a distinct reduction in phosphorylation was found after 24 h of treatment (Fig. 6). Upon incubation with CGRP8–37, PD98059, SB203580, or SP60012 (10^− 6^ M) for 24 and 48 h, the reduction in phosphorylation induced by CGRP was weakened or completely blocked. p-ERK1/2, p-p38, and p-JNK expression were distinctly and steadily increased by 2.02-, 2.44- and 1.54-fold at 24 h, and by 1.44-, 1.25- and 1.58-fold at 48 h, respectively, in the CGRP8–37 group compared with the CGRP-treated group. p-ERK1/2 expression was increased by 1.13- and 1.11-fold at 24 and 48 h, respectively, in the PD98059 group compared with the CGRP-treated group. p-p38 expression was increased by 1.71- and 1.15-fold at 24 and 48 h, respectively, in the SB203580 group compared with the CGRP-treated group. p-JNK expression was increased by 0.77- and 1.26-fold at 24 and 48 h, respectively, in the SP60012 group compared with the CGRP-treated group. These results further indicate the contribution of MAPKs to SD-induced apoptosis.

## Discussion

Neovascularization may emerge as a promising approach for bone regeneration and treatment of ischaemic tissues or blood vessel disorders [34, 35]. EPCs have been demonstrated to be the primary stem cells that activate angiogenesis [6–9]. However, EPC transplantation requires a large number of cells (1.0 × 10^6^–3.0 × 10^6^/kg), which is an unavoidable issue commonly encountered in clinical applications. The number of EPCs as well as their proliferative potential may be reduced in the presence of cardiovascular disease, diabetes mellitus and chronic smoking [36]. Fortunately, in this study, we found that CGRP exerts pro-proliferative and anti-apoptotic effects on EPCs; thus, CGRP can significantly increase the number and the viability of EPCs. In addition, CGRP treatment upregulated cyclin D1 and cyclin E gene expression levels and downregulated the gene or protein levels of apoptosis-related factors. Moreover, these changes might be associated with MAPK signalling.

In our study, we primarily aimed to evaluate the most effective CGRP concentration, which slightly differed among different cells and under in vitro vs. in vivo conditions. A previous study by Haegerstrand A et al. indicated that CGRP induced a concentration-dependent increase in the number of EPCs derived from veins, with a maximal effect at 10^− 8^ M after 4 days of stimulation [22]. A similar study by Mapp et al. indicated that CGRP induced endothelial cell proliferation at a dose of 2.5 × 10^− 9^ M but did not induce significant effects at a dose of 2.5 × 10^− 11^ M in vivo [37]. In our study, we found that all concentrations of CGRP increased the optical density and upregulated cyclin D1 and E mRNA, with the peak effect at 10^− 10^ M. Interestingly, upregulation of cyclin D1 and cyclin E mRNA was observed at 24 and 72 h but not at 48 h.

Our previous study showed that 10^− 10^–10^− 11^ M CGRP significantly increased tube formation in human umbilical vein endothelial cells (HUVECs). More importantly, CGRP promoted EPC proliferation by accelerating G1/S phase transition and upregulating cyclin D1 and cyclin E expression in EPCs, similar to the effects of granulocyte-macrophage colony-stimulating factor (GM-CSF) on EPCs [26].

Apoptosis is involved in regulating cell number under certain physiological and pathological conditions. SD induces apoptosis in EPCs and simulates ischaemic-hypoxic conditions at fracture sites [38]. To our knowledge, this is the first study to report the anti-apoptotic effects of CGRP on EPCs, and the findings expand upon previous reports indicating that CGRP exerts a proliferative effect on angiotensin II-induced EPC senescence [14, 39] and acts as a survival factor in rat gubernaculum [40]. A number of molecules have been identified that either induce or prevent apoptosis. For example, caspase-3 is a crucial component of the final pathway that induces apoptosis [30, 41], which is activated by two initiator pathways driven by caspase-9 and caspase-8 [30]. We found that caspase-3, caspase-8 and caspase-9 mRNA expression levels were remarkably reduced, illustrating anti-apoptotic effects of CGRP on intrinsic and extrinsic cell apoptosis or death pathways in EPCs. An increased Bcl-2/Bax ratio may play an important role in protecting against cell apoptosis [41, 42], and we found that treatment with CGRP resulted in an increase in the level of the anti-apoptotic molecule Bcl-2 and a decrease in the level of the pro-apoptotic molecule Bax, leading to a significantly increased Bcl-2/Bax ratio.

We then investigated potential signalling molecules associated with CGRP-induced EPC proliferation and apoptosis. The results showed that the CGRP-induced increases in proliferation and decreases in apoptosis were completely blocked by preincubation with CGRP8–37, PD98059, SB203580, or SP600125. These findings suggest that CGRP is highly associated with CRLR and MAPK signalling during proliferation and SD-induced apoptosis in EPCs. Several reports have been published regarding the regulation of proliferation and apoptosis by MAPK signalling in other cell types [25–28, 31], although the pathways controlling apoptosis activation and inhibition are inconsistent. p38 functions in an anti-apoptotic pathway, and the p38 pathway inhibitor SB203580 further increased oxidized low-density lipoprotein (oxLDL)-induced apoptosis in EPCs [25]. However, inhibition (preincubation of EPCs with SB20358 for 20 min) of the p38 pathway abolished the glucose-induced reduction in the number of EPCs and partially restored their function, and the ERK activation inhibitor PD98059 had no effect on the number of EPCs [31]. A review by Snigdha et al. noted that ERK1/2 activation has been linked to cell protection against apoptosis, whereas JNK and p38 pathways are often associated with apoptosis induction in response to extracellular stimuli [30]. Here, we show that CGRP induced EPC proliferation and protected EPCs against SD-induced apoptosis possibly by inhibiting the MAPK pathway, although the actual roles of each MAPK cascade highly differ among cell types and are differentially affected by extracellular stimuli and the environment. Cell survival following H_2_O_2_ exposure was affected in three cell lines upon either activation or suppression of the ERK pathway, although the targets of H_2_O_2_ were unknown [43].

In summary, we have shown that CGRP promoted proliferation and inhibited SD-induced apoptosis in a dose- and time-dependent manner. More importantly, CGRP may exert pro-proliferative and anti-apoptotic effects by suppressing the MAPK activation pathway. Thus, we reveal a novel mechanism by which CGRP improves the viability of EPCs, which play a role in vascularization to stimulate fracture healing.

## Additional files

Additional file 1: Antibody against phospho-ERK1/2 instruction. (PDF 451 kb)

Additional file 2: Antibody against phospho-JNK instruction. (PDF 158 kb)

Additional file 3: EPCs identification. (TIF 15332 kb)

## Abbreviations

BMSCs: Bone marrow stromal cell
CCK-8: Cell Counting Kit-8
CGRP: Calcitonin gene-related peptide
CRLR: Calcitonin receptor-like receptor
DAPI: 4′-6′-diamidino-2-phenylindole
EPCs: Endothelial progenitor cells
ERK: Extracellular signal regulated kinase
FBS: Fetal bovine serum
FITC: Fluorescein isothiocyanate
JNK: c-Jun N-terminal kinase
MAPK: Mitogen-activated protein kinases
PBS: Phosphate buffer solution
q-PCR: Quantitative polymerase chain reaction
