# Supplementary figures and images for: Calcitonin gene-related peptide promotes proliferation and inhibits apoptosis in endothelial progenitor cells via inhibiting MAPK signaling
Source: Proteome Sci. 2018 Nov 14;16:18. doi: 10.1186/s12953-018-0146-4 (PMC6236989; doi:10.1186/s12953-018-0146-4)

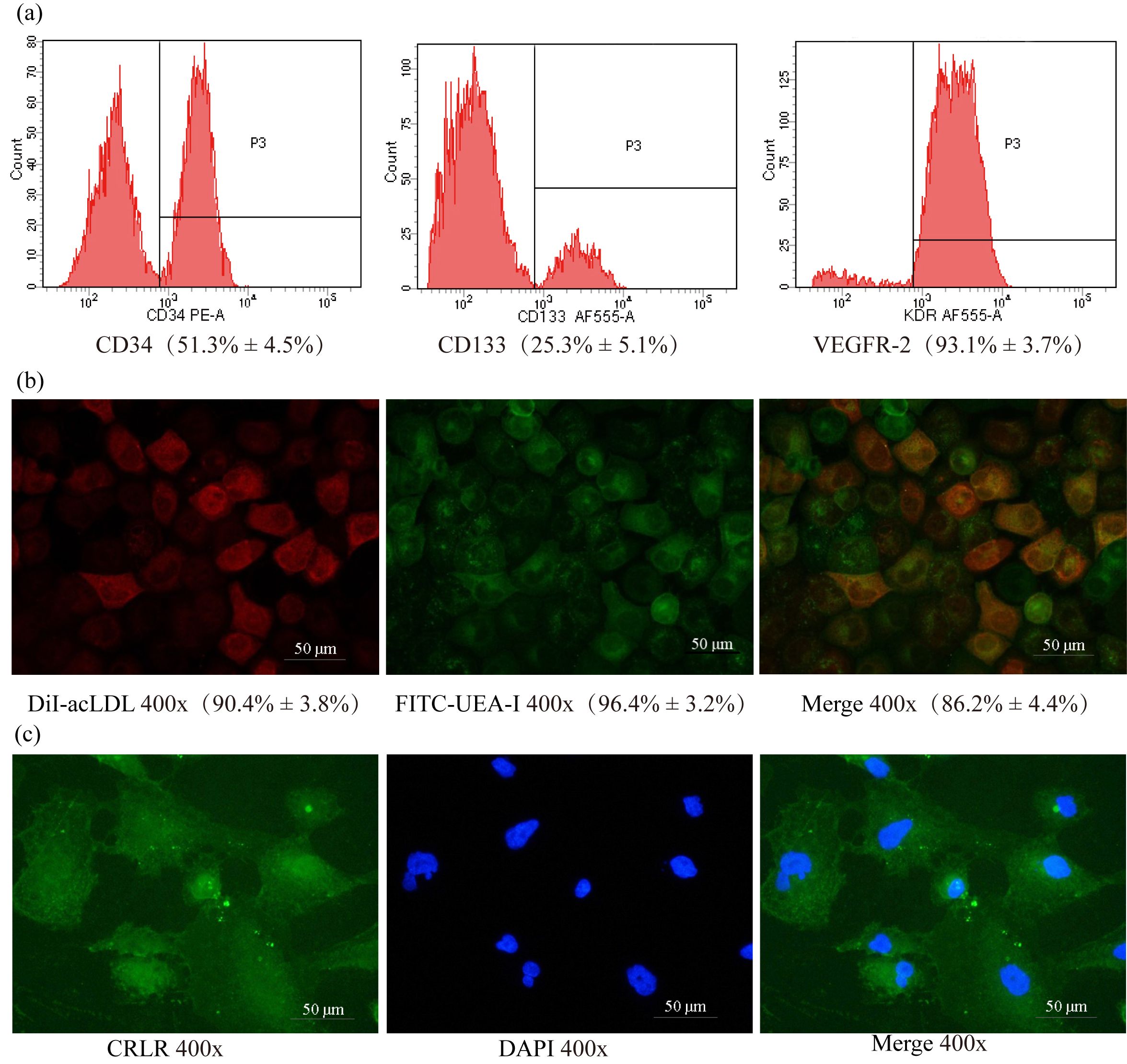

Supplement: Supplementary file 1 — Antibody against phospho-ERK1/2 instruction. (PDF 451 kb) [file 12953_2018_146_MOESM1_ESM.tif]
