# Supplementary material for: Calcitonin gene-related peptide promotes proliferation and inhibits apoptosis in endothelial progenitor cells via inhibiting MAPK signaling
Source: Proteome Sci. 2018 Nov 14;16:18. doi: 10.1186/s12953-018-0146-4 (PMC6236989; doi:10.1186/s12953-018-0146-4)
Supplement: Supplementary file 2 — Antibody against phospho-JNK instruction. (PDF 158 kb) [file 12953_2018_146_MOESM2_ESM.pdf]

#4370 Store at –20°C

Phospho-p44/42 MAPK (Erk1/2) (Thr202/Tyr204) (D13.14.4E) XP® Rabbit mAb

Cell Signaling TECHNOLOGY®

Orders ■ 877-616-CELL (2355)
orders@cellsignal.com
Support ■ 877-678-TECH (8324)
info@cellsignal.com
Web ■ www.cellsignal.com

rev. 12/04/17

For Research Use Only. Not For Use In Diagnostic Procedures.

Entrez-Gene ID # 5595, 5594
Swiss-Prot Acc. # P27361, P28482

Applications	Species Cross-Reactivity*	Molecular Wt.	Isotype
W, IP, IHC-P, IF-IC, F Endogenous	H, M, R, Mk, Mi, Pg, Sc, Hm, B, Dm, Z, Dg, (C,Ce)	44, 42 kDa	Rabbit IgG**

Background: Mitogen-activated protein kinases (MAPKs) are a widely conserved family of serine/threonine protein kinases involved in many cellular programs such as cell proliferation, differentiation, motility, and death. The p44/42 MAPK (ERK1/2) signaling pathway can be activated in response to a diverse range of extracellular stimuli including mitogens, growth factors, and cytokines (1-3) and is an important target in the diagnosis and treatment of cancer (4). Upon stimulation, a sequential three-part protein kinase cascade is initiated, consisting of a MAP kinase kinase kinase (MAPKKK), a MAP kinase kinase (MAPKK), and a MAP kinase. While multiple ERK1/2 MAP3Ks have been identified, including the Raf family, Mos, and Tpl2/Cot, MEK1 and MEK2 are the primary MAPKKs in this pathway (5,6). MEK1 and MEK2 activate ERK1/p44 and ERK2/p42 through phosphorylation of activation loop residues Thr202/Tyr204 and Thr185/Tyr187, respectively. Several downstream targets of ERK1/2 have been identified, including p90RSK (7) and the transcription factor Elk-1 (8,9). ERK1/2 are negatively regulated by a family of dual-specificity (Thr/Tyr) MAPK phosphatases, known as DUSPs or MKPs (10), along with MEK inhibitors such as U0126 and PD98059.

Specificity/Sensitivity: Phospho-p44/42 MAPK (Erk1/2) (Thr202/Tyr204) (D13.14.4E) XP® Rabbit mAb detects endogenous levels of p44 and p42 MAP Kinase (Erk1 and Erk2) when dually phosphorylated at Thr202 and Tyr204 of Erk1 (Thr185 and Tyr187 of Erk2), and singly phosphorylated at Thr202. This antibody does not cross-react with the corresponding phosphorylated residues of either JNK/SAPK or p38 MAP kinases.

Source/Purification: Monoclonal antibody is produced by immunizing animals with a synthetic phosphopeptide corresponding to residues surrounding Thr202/Tyr204 of human p44 MAP kinase.

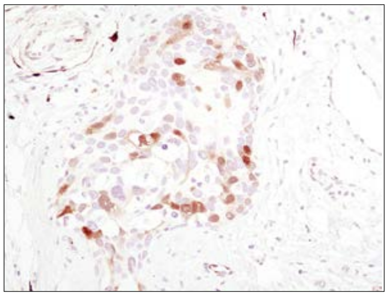

Immunohistochemical analysis of paraffin-embedded human breast carcinoma using Phospho-p44/42 MAPK (Erk1/2) (Thr202/Tyr204) (D13.14.4E) XP® Rabbit mAb.

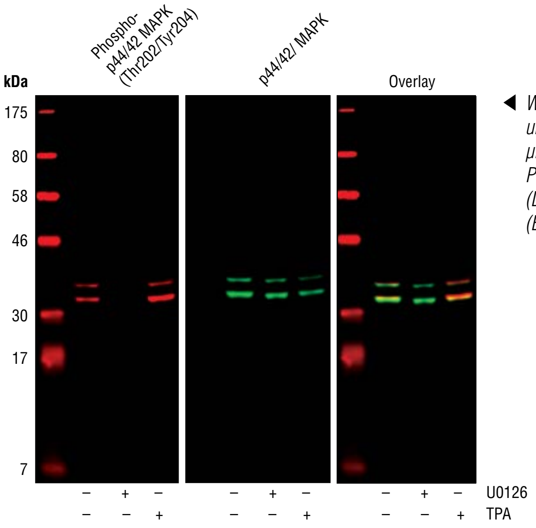

◀ *Western blot analysis of extracts from COS cells, untreated or treated with either U0126 #9903 (10 μM for 1h) or TPA #9905 (200 nM for 10m), using Phospho-p44/42 MAPK (Erk1/2) (Thr202/Tyr204) (D13.14.4E) XP® Rabbit mAb #4370 and p44/42 MAPK (Erk1/2) (3A7) Mouse mAb #9107.*

Storage: Supplied in 10 mM sodium HEPES (pH 7.5), 150 mM NaCl, 100 µg/ml BSA, 50% glycerol and less than 0.02% sodium azide. Store at –20°C. *Do not aliquot the antibody.*

***Species cross-reactivity is determined by western blot.**

****Anti-rabbit secondary antibodies must be used to detect this antibody.**

Recommended Antibody Dilutions:

Western blotting	1:2000
Immunoprecipitation	1:50
Immunohistochemistry (Paraffin)	1:400†
Unmasking buffer:	Citrate
Antibody diluent:	SignalStain® Antibody Diluent #8112
Detection reagent:	SignalStain® Boost (HRP, Rabbit) #8114
†*Optimal IHC dilutions determined using SignalStain® Boost IHC Detection Reagent.*	
Immunofluorescence (IF-IC)	1:200
IF Protocol:	Methanol Permeabilization Required
Flow Cytometry:	1:800

For application specific protocols please see the web page for this product at www.cellsignal.com.

Please visit www.cellsignal.com for a complete listing of recommended companion products.

IMPORTANT: For western blots, incubate membrane with diluted antibody in 5% w/v BSA, 1X TBS, 0.1% Tween-20 at 4°C with gentle shaking, overnight.

Alexa Fluor® is a registered trademark of Life Technologies Corporation.

DRAQ5® is a registered trademark of Biostatus Limited.

© 2014 Cell Signaling Technology, Inc.
Cell Signaling Technology® is a trademark of Cell Signaling Technology, Inc.

Applications Key: **W**—Western **IP**—Immunoprecipitation **IHC**—Immunohistochemistry **ChIP**—Chromatin Immunoprecipitation **IF**—Immunofluorescence **F**—Flow cytometry **E-P**—ELISA-Peptide

Species Cross-Reactivity Key: **H**—human **M**—mouse **R**—rat **Hm**—hamster **Mk**—monkey **Mi**—mink **C**—chicken **Dm**—D. melanogaster **X**—Xenopus **Z**—zebrafish **B**—bovine **Dg**—dog **Pg**—pig **Sc**—S. cerevisiae **Ce**—C. elegans **Hr**—Horse **All**—all species expected Species enclosed in parentheses are predicted to react based on 100% homology.

#4370

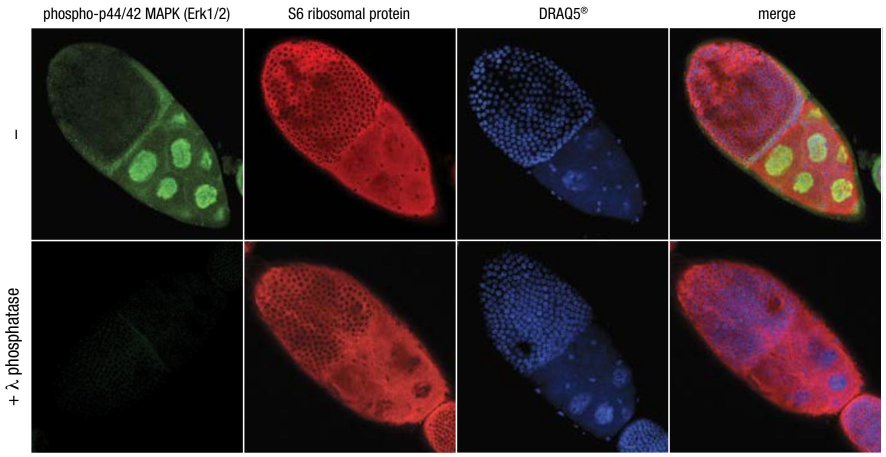

Confocal immunofluorescent analysis of Drosophila egg chambers, untreated (top) or λ phosphatase-treated (bottom), using Phospho-p44/42 MAPK (Erk1/2) (Thr202/Tyr204) (D13.14.4E) XP® Rabbit mAb #4370 (green) and S6 Ribosomal Protein (54D2) Mouse mAb #2317 (red). Blue pseudocolor = DRAQ5® #4084 (fluorescent DNA dye).

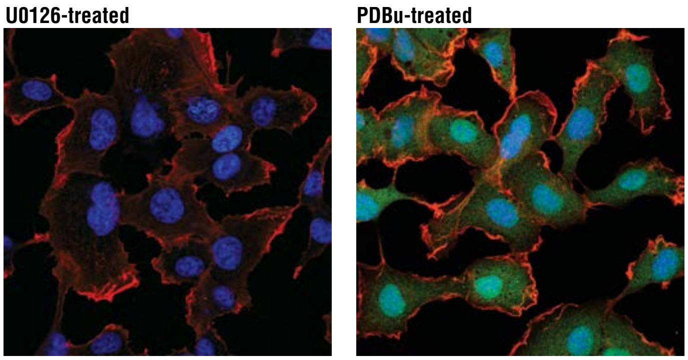

Confocal immunofluorescent analysis of HT1080 cells, starved overnight then treated with U0126 #9903 (10 uM, 2 h; left) or PDBu (Phorbol 12,13-Dibutyrate) #12808 (100 nM, 15 m; right) using Phospho-p44/42 MAPK (Erk1/2) (Thr202/Tyr204) (D13.14.4E) XP® Rabbit mAb #4370 (green) and β-Actin (8H10D10) Mouse mAb #3700 (red). Blue pseudocolor = DRAQ5® #4084 (fluorescent DNA dye).

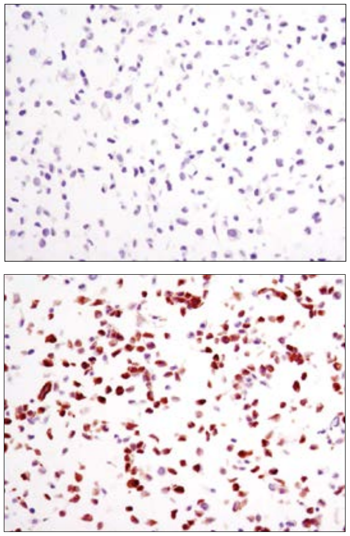

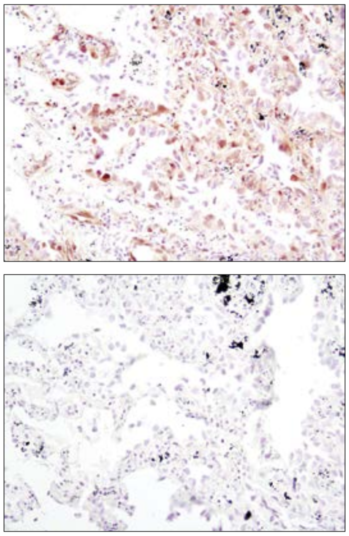

Immunohistochemical analysis using Phospho-p44/42 MAPK (Erk1/2) (Thr202/Tyr204) (D13.14.4E) XP® Rabbit mAb on SignalSlide™ Phospho-p44/42 MAPK (Thr202/Tyr204) IHC Controls #8103 (paraffin-embedded NIH/3T3 cells, treated with U0126 #9903 (upper) or TPA #4174 (lower).

Immunohistochemical analysis of paraffin-embedded human lung carcinoma, untreated (upper) or λ phosphatase-treated (lower), using Phospho-p44/42 MAPK (Erk1/2) (Thr202/Tyr204) (D13.14.4E) XP® Rabbit mAb.

Background References:

(1) Roux, P.P. and Blenis, J. (2004) *Microbiol Mol Biol Rev* 68, 320–44.

(2) Baccarini, M. (2005) *FEBS Lett* 579, 3271–7.

(3) Meloche, S. and Pouysségur, J. (2007) *Oncogene* 26, 3227–39.

(4) Roberts, P.J. and Der, C.J. (2007) *Oncogene* 26, 3291–310.

(5) Rubinfeld, H. and Seger, R. (2005) *Mol Biotechnol* 31, 151–74.

(6) Murphy, L.O. and Blenis, J. (2006) *Trends Biochem Sci* 31, 268–75.

(7) Dalby, K.N. et al. (1998) *J Biol Chem* 273, 1496–505.

(8) Marais, R. et al. (1993) *Cell* 73, 381–93.

(9) Kortenjann, M. et al. (1994) *Mol Cell Biol* 14, 4815–24.

(10) Owens, D.M. and Keyse, S.M. (2007) *Oncogene* 26, 3203–13.

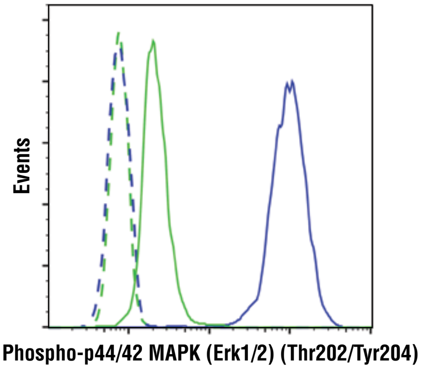

Flow cytometric analysis of Jurkat cells, treated with U0126 (10uM, 2 hrs; blue) or treated with TPA #4174 (200nM, 30 min; green) using Phospho-p44/42 MAPK (Erk1/2) (Thr202/Tyr204) (D13.14.4E) XP® Rabbit mAb (solid lines) or concentration-matched Rabbit (DA1E) mAb IgG XP® Isotype Control #3900 (dashed lines). Anti-rabbit IgG (H+L), F(ab')2 Fragment (Alexa Fluor® 488 Conjugate) #4412.

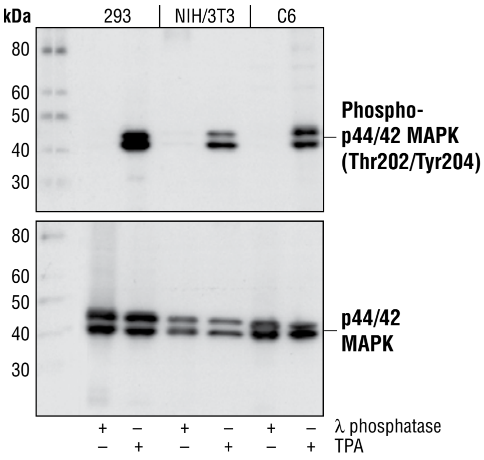

Western blot analysis of extracts from 293, NIH/3T3 and C6 cells, treated with λ phosphatase or TPA as indicated, using Phospho-p44/42 MAPK (Erk1/2) (Thr202/Tyr204) (D13.14.4E) XP® Rabbit mAb (upper), or p44/42 MAPK (Erk1/2) (137F5) Rabbit mAb #4695 (lower).

© 2011 Cell Signaling Technology, Inc.
