# Supplementary material for: Calcitonin gene-related peptide promotes proliferation and inhibits apoptosis in endothelial progenitor cells via inhibiting MAPK signaling
Source: Proteome Sci. 2018 Nov 14;16:18. doi: 10.1186/s12953-018-0146-4 (PMC6236989; doi:10.1186/s12953-018-0146-4)
Supplement: Supplementary file 3 — EPCs identification. (TIF 15332 kb) [file 12953_2018_146_MOESM3_ESM.pdf]

Phospho-SAPK/JNK (Thr183/Tyr185) (G9) Mouse mAb

#9255 Store at -20°C

Cell Signaling TECHNOLOGY®

Orders ■ 877-616-CELL (2355)
orders@cellsignal.com
Support ■ 877-678-TECH (8324)
info@cellsignal.com
Web ■ www.cellsignal.com

rev. 05/16/18

For Research Use Only. Not For Use In Diagnostic Procedures.

Entrez-Gene ID #5599
Swiss-Prot Acc. #P45983

Applications	Species Cross-Reactivity*	Molecular Wt.	Isotype
W, IP, IF-IC, F Endogenous	H, M, R, Hm, Sc	46 kDa, 54 kDa	Mouse IgG1**

Background: The stress-activated protein kinase/Jun-amino-terminal kinase SAPK/JNK is potently and preferentially activated by a variety of environmental stresses including UV and gamma radiation, ceramides, inflammatory cytokines and in some instances, by growth factors and GPCR agonists (1-6). As with the other MAPKs, the core signaling unit is composed of a MAPKKK, typically MEKK1-MEKK4, or by one of the mixed lineage kinases (MLKs), which phosphorylate and activate MKK4/7. Upon activation, MKKs phosphorylate and activate the SAPK/JNK kinase (2). Stress signals are delivered to this cascade by small GTPases of the Rho family (Rac, Rho, cdc42) (3). Both Rac1 and cdc42 mediate the stimulation of MEKKs and MLKs (3). Alternatively, MKK4/7 can be activated in a GTPase independent mechanism via stimulation of a germinal center kinase (GCK) family member (4). There are three SAPK/JNK genes each of which undergoes alternative splicing resulting in numerous isoforms (3). SAPK/JNK, when active as a dimer, can translocate to the nucleus and regulate transcription through its effects on c-Jun, ATF-2 and other transcription factors (3,5).

Specificity/Sensitivity: Phospho-SAPK/JNK (Thr183/Tyr185) (G9) Mouse mAb detects endogenous levels of p46 and p54 SAPK/JNK dually phosphorylated at Thr183 and Tyr185. This antibody does not recognize endogenous levels of phosphorylated p44/42 MAPK or p38 MAP kinase.

Source/Purification: Monoclonal antibody is produced by immunizing animals with a synthetic phosphopeptide corresponding to residues surrounding Thr183/Tyr185 of human SAPK/JNK.

Background References:

(1) Davis, R.J. (1999) *Biochem Soc Symp* 64, 1-12.
(2) Ichijo, H. (1999) *Oncogene* 18, 6087-93.
(3) Kyriakis, J.M. and Avruch, J. (2001) *Physiol Rev* 81, 807-69.
(4) Kyriakis, J.M. (1999) *J Biol Chem* 274, 5259-62.
(5) Leppä, S. and Bohmann, D. (1999) *Oncogene* 18, 6158-62.
(6) Whitmarsh, A.J. and Davis, R.J. (1998) *Trends Biochem Sci* 23, 481-5.

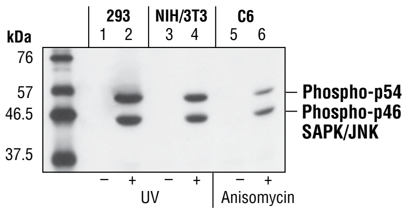

Western blot analysis of extracts from 293 cells, untreated or UV-treated (lanes 1 and 2), NIH/3T3 cells, untreated or UV-treated (lanes 3 and 4) and C6 cells, untreated or anisomycin-treated (lanes 5 and 6), using Phospho-SAPK/JNK (Thr183/Tyr185) (G9) Mouse mAb.

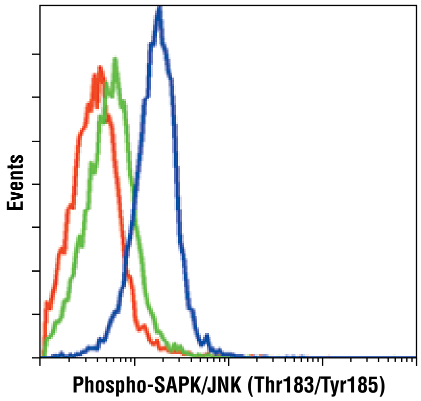

Flow cytometric analysis of Jurkat cells, untreated (green) or anisomycin-treated (blue), using Phospho-SAPK/JNK (Thr183/Tyr185) (G9) Mouse mAb compared to a nonspecific negative control antibody (red).

Storage: Supplied in 10 mM sodium HEPES (pH 7.5), 150 mM NaCl, 100 µg/ml BSA, 50% glycerol and less than 0.02% sodium azide. Store at –20°C. *Do not aliquot the antibody.*

***Species cross-reactivity is determined by western blot.**

****Anti-mouse secondary antibodies must be used to detect this antibody.**

Recommended Antibody Dilutions:

Western Blotting	1:2000
Immunoprecipitation	1:250
Flow Cytometry	1:400
Immunofluorescence (IF-IC)	1:200

For application specific protocols please see the web page for this product at www.cellsignal.com.

Please visit www.cellsignal.com for a complete listing of recommended companion products.

IMPORTANT: For western blots, incubate membrane with diluted antibody in 5% w/v nonfat dry milk, 1X TBS, 0.1% Tween-20 at 4°C with gentle shaking, overnight.

Applications Key: **W**—Western **IP**—Immunoprecipitation **IHC**—Immunohistochemistry **ChIP**—Chromatin Immunoprecipitation **IF**—Immunofluorescence **F**—Flow cytometry **E-P**—ELISA-Peptide

Species Cross-Reactivity Key: **H**—human **M**—mouse **R**—rat **Hm**—hamster **Mk**—monkey **Mi**—mink **C**—chicken **Dm**—D. melanogaster **X**—Xenopus **Z**—zebrafish **B**—bovine **Dg**—dog **Pg**—pig **Sc**—S. cerevisiae **Ce**—C. elegans **Hr**—horse **All**—all species expected Species enclosed in parentheses are predicted to react based on 100% homology.

© 2016 Cell Signaling Technology, Inc.
Cell Signaling Technology® is a trademark of Cell Signaling Technology, Inc.

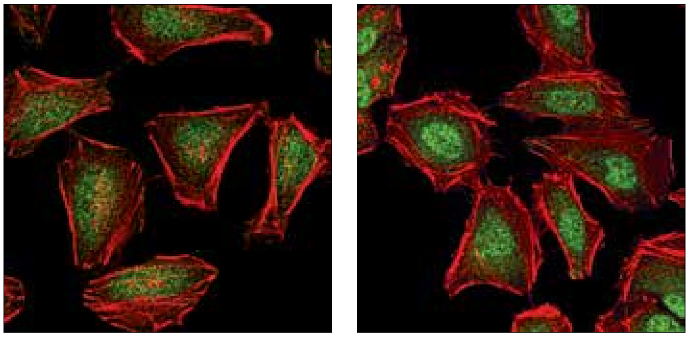

Confocal immunofluorescent analysis of HeLa cells untreated (left) and anisomycin-treated (right) using Phospho-SAPK/JNK (Thr183/Tyr185) (G9) Mouse mAb (green). Actin filaments have been labeled with DY-554 phalloidin (red).

© 2012 Cell Signaling Technology, Inc.
